# *Bacillus atrophaeus* DX-9 biocontrol against potato common scab involves significant changes in the soil microbiome and metabolome

**DOI:** 10.1007/s42994-025-00199-3

**Published:** 2025-02-18

**Authors:** Jingjing Cao, Yue Ma, Jing Fu, Zhiqin Wang, Yonglong Zhao, Naiqin Zhong, Pan Zhao

**Affiliations:** 1 State Key Laboratory of Plant Genomics, Institute of Microbiology, Chinese Academy of Sciences, Beijing, 100101 China; 2 Engineering Laboratory for Advanced Microbial Technology of Agriculture, Chinese Academy of Sciences, Beijing, 100101 China; 3 School of Agriculture, Ningxia University, Yinchuan, 750000 China; 4 Qi Biodesign, Beijing, 100101 China; 5 Key Laboratory of Potato Industry Integration and Development Enterprises in Inner Mongolia Autonomous Region, Hulunbuir, 021000 China

**Keywords:** *Bacillus atrophaeus*, *Solanum tuberosum*, Biocontrol, Common scab, Soil properties, Microbial community

## Abstract

Potato common scab (CS) is a worldwide disease, caused by *Streptomyces* spp., and its presence reduces the market value of potatoes. A nontoxic and potentially effective approach in many control strategies is the use of antagonistic microbes as biocontrol agents. In this study, *Bacillus atrophaeus* DX­9 was isolated and assessed for its ability to protect against CS. Through integrated metagenomic and metabolomic analyses, changes in the soil microbial community structure and soil properties were analyzed to understand the effects of *Bacillus atrophaeus* DX­9 on CS. These studies revealed that DX­9 inoculation could significantly decrease CS disease rate, disease index, and the number of CS pathogens, along with an increase in soil N and P content. Our metagenomic assays identified 102 phyla and 1154 genera, and DX­9 inoculation increased the relative abundances of the phyla Pseudomonadota, Chloroflexota and Gemmatimonadota. Additionally, an increase in the relative abundance of genera, such as *Bradyrhizobium*, *Agrobacterium*, and *Nitrobacter*, were significantly and positively correlated with soil N and P. Metabolomic analysis revealed that DX­9 inoculation significantly increased the soil levels of phytolaccoside A, 7,8­dihydropteroic acid, novobiocin, and azafrin. These compounds were enriched in microbe pathway metabolites, including xenobiotic biodegradation and metabolism, biosynthesis of other secondary metabolites, and metabolism of cofactors and vitamins. In summary, the use of *Bacillus atrophaeus* DX­9 against potato CS offers an alternative biocontrol method that can improve both soil microbial community and properties. This study provides insight into the potential mechanisms by which microbial inoculants can control CS disease.

**Supplementary Information:**

The online version contains supplementary material available at 10.1007/s42994-025-00199-3.

## Introduction

Potato (*Solanum tuberosum* L.) is an important crop, worldwide, and is the fourth most common food crop in China. In 2022, the potato-growing area in China was more than 5.73 million hectares, and potato production exceeded 95.57 million metric tons (FAOSTAT, http://www.fao.org/faostat/en/#data/QCL/visualize; accessed on February 1, 2024). Potatoes produce more energy and protein than other food crops (David 2009), and they represent a valuable source of carbohydrates, proteins, minerals, and vitamins (Fernie and Willmitzer 2001). During the growth period, numerous diseases, especially common scab (CS), threaten potato yield and quality (Braun et al. 2017). CS in potato tubers results in skin lesions and affects tuber quality, reducing their market value. In recent years, CS has caused substantial economic losses, worldwide, and has become a major concern for potato farmers (Zhao et al. 2022).

CS is caused by species of the soilborne, Gram‐positive, filamentous bacterial genus *Streptomyces*. The *Streptomyces* pathogens that have been reported to cause CS include *S. scabiei*, *S. acidiscabies*, *S. turgidiscabies*, and *S. europaeiscabiei* (Sarwar et al. 2018). Among these pathogens, *S. scabiei* is the most important and best‐characterized. It secretes a phytotoxin called thaxtomin A (TA), which results in CS in potatoes (Loria et al. 2008). TA is synthesized by a nonribosomal peptide synthetase (NRPS), which is encoded by the *txtA* and *txtB* genes (Healy et al. 2000). Additionally, a P450 monooxygenase (encoded by *txtC*) promotes post-cyclization hydroxylation, a nitric oxide synthase (encoded by *nos/txtD*) is responsible for dipeptide nitration (Kers et al. 2004), and a cytochrome P450 (encoded by *txtE*) catalyzes site-specific nitration (Barry et al. 2012). These genes for TA biosynthesis are clustered on a pathogenicity island (PAI) in pathogen genomes.

Given the increasing incidence and economic losses related to CS, many control strategies are used to reduce CS, including irrigation during tuber initiation, crop rotation, soil fumigation, soil pH modification, foliar sprays, and the use of disease-resistant cultivars (Dees and Wanner 2012). However, these measures are not very effective at controlling CS. For a long time, chemical methods were the main control treatments, but are expensive, pollute the environment, and negatively affect soil microbes (Kobayashi et al. 2015). Many inexpensive, nontoxic strategies are now available for controlling CS. For example, foliar sprays of auxins and related molecules, including 2,4­dichlorophenoxyacetic acid (2,4­D), indole­3­acetic acid (IAA), benzothiadiazole (BTH), β­aminobutyric acid (BABA), acetylsalicylic acid (ASA), and tryptophan, trigger the host immune response to protect against CS (Zhao et al. 2022).

Moreover, one of the most attractive methods is biological control. Nonpathogenic *Streptomyces* species control CS by antibiotic production, extracellular enzyme secretion and competition (Eckwall and Schottel 1997). *Streptomyces* A1RT controls CS by promoting plant growth (Sarwar et al. 2018). The *Streptomyces* sp. isolate WoRs-501 decreases the abundance of CS pathogens by synthesizing antibiotics (Kobayashi et al. 2015). Some nonpathogenic *Streptomyces* species have also been shown to have dual effects, which involve controlling CS and increasing the potato yield. For example, *S. violaceusniger* AC12AB has dual effects, as it can decrease CS severity and increase the potato yield (Sarwar et al. 2019). Additionally, *Pseudomonas* produces phenazine­1­carboxylic acid to inhibit *S. scabiei* growth (St-Onge et al. 2011). Furthermore, *Bacillu*s sp. sunhua significantly decreased the CS pathogen infection rate in a pot assay (Han et al. 2005), and *B. laterosporus* AMCC100017 steadily colonized the rhizosphere soil and significantly inhibited the CS pathogen (Chen et al. 2017). Species in the fungal phylum Ascomycota (such as *Trichoderma*) also have reported antagonistic effects against CS pathogens, especially at low pH (Tagawa et al. 2010). Other antagonistic microbes, such as *Trichoderma*, *Enterobacter*, *Acinetobacter*, and bacteriophages, may also be useful for controlling CS (McKenna et al. 2001).

The biocontrol of CS, by inoculation with antagonistic microbes, can impact soil properties, the local microbial community, and plant growth. To understand the association between microbial inoculation and reduced CS, it is necessary to study the changes in the soil microbial community and soil metabolites. Most soil microbes cannot be cultured, but high-throughput sequencing has allowed previously unknown microbes to be explored (Thompson et al. 2017). Metagenomics reveals the genetic diversity of all microbial material in a biological sample (including unculturable microbes) (Aguiar et al. 2016), but it only provides an overview of potential microbial functions. The actual functions need to be determined by other methods (Jansson and Hofmockel 2018). Metabolomics is an approach for studying all metabolites in a biological sample, understanding metabolic networks, and comparing metabolite levels among biological samples (Palazzotto and Weber 2018). Soil metabolomics can directly reveal the biological responses of soil microbes to different conditions (Song et al. 2020).

There is a lack of multi-omics studies on changes in the soil microbial community after antagonistic microbe inoculation to control CS. This limits our understanding of the soil dynamics related to CS control by microbial inoculation (such as *Bacillus atrophaeus* DX-9 inoculation). In this study, we assessed the effects of *B. atrophaeus* DX­9 inoculation on CS control, the soil microbial community, and soil metabolites. An integrated metagenomic and metabolomic analysis was used to investigate the changes in the soil microbial community and soil metabolites. These results helped to establish a connection between the soil microbiome and CS, after DX­9 inoculation, offering a mechanistic explanation of the effects of *Bacillus atrophaeus* DX-9, and advanced our knowledge regarding this CS biocontrol strategy.

## Results

### Screening and identification of the antagonistic bacterial strain DX-9

Among the five bacterial isolates that exhibited the highest antagonistic activity against *S. scabiei* 4.1765, the isolate designated DX­9 had the strongest antagonistic activity, with an inhibition zone diameter of 40.3 mm. Additionally, five other phytopathogens (including *Erwinia carotovora* subsp*. carotovora*, *Rhizoctonia solani*, *Fusarium oxysporum*, *Alternaria solani* and *Verticillium dahliae*) were used to test whether DX-9 has broad-spectrum antagonistic activity. These results confirmed that DX-9 had broad-spectrum antagonistic activity (Fig. S1). Therefore, DX­9 was preserved in our laboratory and in the China General Microbiological Culture Collection (CGMCC no. 21600).

DX­9 was identified as a *Bacillus* species using 16*S* rRNA sequence and phylogenetic tree analyses; it exhibited 99.93% homology with the *B. atrophaeus* strain KT719435 (Fig. S2). Thus, DX-9 was identified and named as *B. atrophaeus* DX­9. Based on our serial dilution experiments, 1 × 10^–9^ cfu/mL DX­9 was identified as the minimum concentration that produced a large inhibition zone diameter. Therefore, this concentration was selected for the pot assays.

### Effects of DX­9 inoculation on CS

**Fig. 1:**
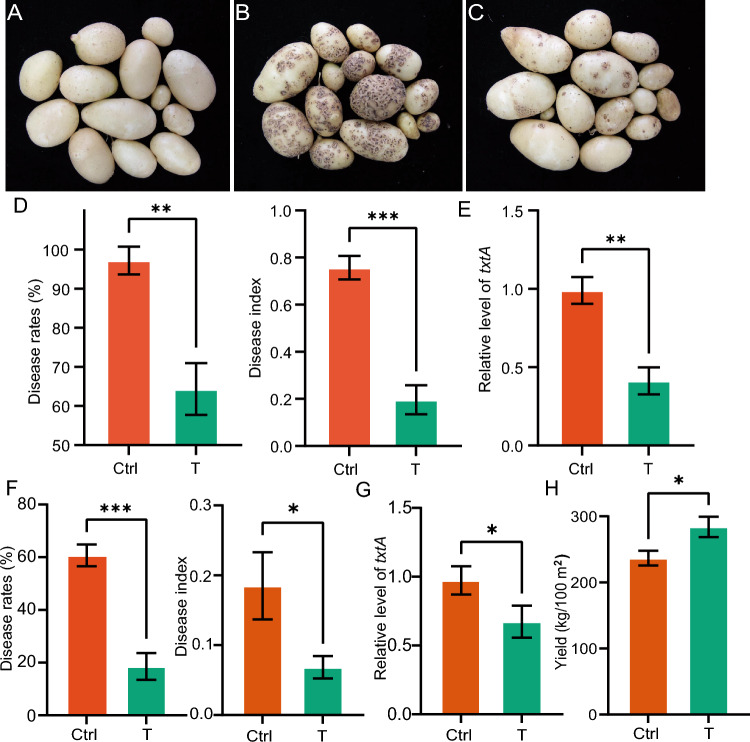
Effects of DX-9 on potato CS. **A–C** Disease symptoms of potatoes assessed by pot assays. **A** Uninoculated plants. **B** Ctrl (control), plants inoculated with *S. scabiei* 4.1765. **C** T (treatment), plants co-inoculated with *S. scabiei* 4.1765 and DX­9. **D** CS disease rates and disease index of potatoes harvested from pots. **E** Relative level of *txtA* in potatoes harvested from pots. **F** CS disease rates and disease index of potatoes harvested from the field. **G** Relative level of *txtA* in potatoes harvested from the field. **H** Yield of DX-9 in the field. Data are presented as mean ± SD (*P < 0.05, **P < 0.01, ***P < 0.001, based on Student’s *t* test)

The control efficacy of DX­9 against potato CS was assessed in pot assays and field trials. In the pot assays, *S. scabiei* 4.1765 inoculation caused lesions on the tuber surfaces, indicating that *S. scabiei* 4.1765 inoculation was very effective (Fig. 1A–C). Co-inoculation with DX­9 led to fewer and smaller tuber lesions (Fig. 1C).

Next, the disease rates, disease index, and control efficacy in the pot assays were determined. The disease rate decreased from 97.2% in the Ctrl (control) group to 64.3% in the T (treatment) group (Fig. 1D). The disease index decreased from 75.7 in the Ctrl group to 19.7 in the T group (Fig. 1D). Finally, the control efficacy of the T group compared with the Ctrl group was 74.1%. Furthermore, to assess the number of pathogenic *Streptomyces* bacteria in the soil, the relative level of *txtA* was determined by quantitative real time polymerase chain reaction (qRT-PCR). The relative level of *txtA* was significantly lower in the T group than in the Ctrl group (Fig. 1E). Thus, there was a larger population of *S. scabiei* 4.1765 in the Ctrl group than in the T group. The field trial results were consistent with these pot assay results. The disease rate decreased from 60.9% in the Ctrl group to 18.0% in the T group, and the disease index decreased from 0.18 in the Ctrl group to 0.07 in the T group (Fig. 1F). The control efficacy of the T group compared with that of the Ctrl group was 61.1%. The relative level of *txtA* was also significantly lower in the T group than in the Ctrl group (Fig. 1G). Next, we compared the potato yield in the field in the two groups, as many biocontrol bacteria have plant growth-promoting effects. These results showed that the potato yield was significantly higher in the T group than the Ctrl group (Fig. 1H).

These data indicated that DX­9 inoculation can significantly decrease the number of CS pathogens in the soil and have a beneficial disease-suppressive effect on potato CS.

### Effects of DX-9 inoculation on soil properties

Beneficial microbial inoculants can protect plants against pathogens and improve soil quality. Therefore, we next analyzed the changes in the soil chemical properties after DX­9 inoculation, including pH, organic matter (OM), total nitrogen (TN), total phosphorus (TP), total potassium (TK), available nitrogen (AN), available phosphorus (AP), and available potassium (AK) (Table 1). The impact on pH, OM, and TK was not significant, whereas the increase for TN, TP, AN, and AP was significant. The AK in the T soil decreased compared with the Ctrl soil, but this change was not significant. These changes in the soil properties revealed that DX­9 could improve soil quality.

**Table 1:** Soil properties in the Ctrl and T groups

Soil property	Ctrl	T
pH	5.36 ± 0.01	5.39 ± 0.03
OM (g/kg)	44.80 ± 1.70	44.37 ± 2.88
TN (g/kg)	2.04 ± 0.22	2.26 ± 0.09*
TP (mg/kg)	455.33 ± 8.38	497.67 ± 13.82*
TK (g/kg)	2.65 ± 0.15	2.66 ± 0.20
AN (mg/kg)	139.67 ± 6.65	153.33 ± 2.87**
AP (mg/kg)	85.27 ± 7.63	112.00 ± 4.24*
AK (mg/kg)	228.00 ± 26.99	203.00 ± 20.83

^*^P < 0.05, **P < 0.01, based on Student’s *t*­test

### Overview of the microbial community assembly and composition

A total of 118.77 GB of clean data were obtained from the metagenomic sequencing analysis of the T and Ctrl rhizosphere soil samples. After assembly by MEGAHIT, 1,059,075 scaffolds (length > 500 bp; mean length, 724 bp) were obtained from all the samples. In total, 1,482,244 open reading frames (ORFs) (length of 100 bp) were analyzed. There were 682,897 genes common to both the T and Ctrl groups, 46,284 unique genes in the Ctrl group, and 40,248 unique genes in the T group. To study microbial diversity in soil samples, alpha diversity can be used to reflect the abundance and diversity of the microbial community. Our results revealed that the diversity indices (Shannon and Simpson indices) and species richness indices (ACE and Chao1) were not significantly different between the T and Ctrl groups (Table 2), indicating that alpha diversity was not affected by the DX­9 inoculation.

**Table 2:** Alpha diversity in the Ctrl and T groups

Sample_ID	ACE	Chao1	Simpson	Shannon
Ctrl	461,308 ± 9599	449,457 ± 10,931	1	18.37 ± 0.03
T	444,771 ± 13,200	433,580 ± 15,373	1	18.34 ± 0.05

The predicted ORFs were annotated by using DIAMOND (v0.9.22) to conduct BLAST searches against the NCBI-NR database (v2018.01). Thus, the taxonomic annotations and relative abundances of microbes in the T and Ctrl groups were obtained. On the basis of the relative abundances, the majority (74%) of the microbes were bacteria, whereas eukaryotes accounted for only 0.3%. The composition of the microbial communities was compared at the phylum and genus levels, and beta diversity was subsequently analyzed.

**Fig. 2:**
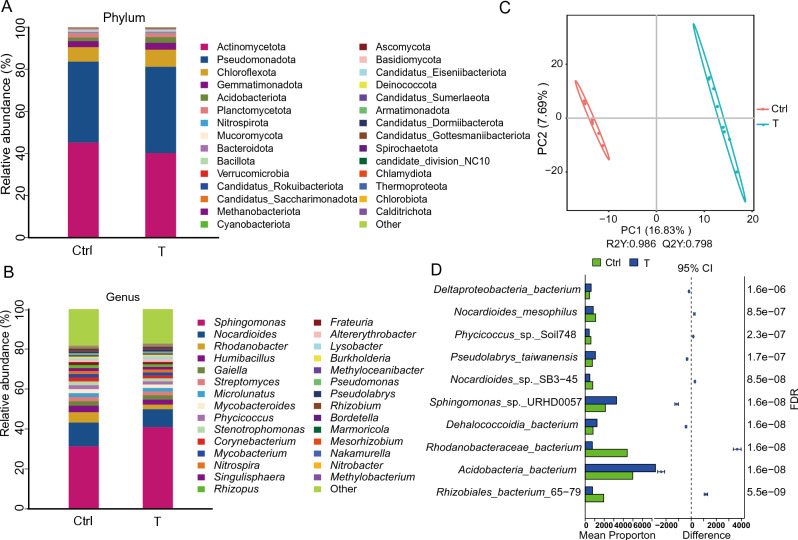
Differences in relative microbial abundances between the T and Ctrl groups. **A** Relative microbial abundance at the phylum level. **B** Relative microbial abundance at the genus level. **C** Partial least squares discriminant analysis (PLS­DA) score plots. **D** Statistical analysis of taxonomic and functional profiles (STAMP) analysis. False discovery rate (FDR) < 0.05

At the phylum level, 102 taxa in the Ctrl and T groups were classified. The dominant phyla in both groups were Actinomycetota (45.2% vs 40.2%), Pseudomonadota (38.4% vs 40.9%), Chloroflexota (6.9% vs 8.2%), Gemmatimonadota (2% vs 3.2%), Acidobacteriota (1.7% vs 2.7%), and Planctomycetota (2.2% vs 2.0%) (Fig. 2A). The Metastats method was used to determine the significant (*P* < 0.05) differences in the relative abundances of phyla between the two groups. With respect to the top six phyla, the relative abundances of Actinomycetota and Planctomycetota were not significantly lower, the relative abundances of Pseudomonadota and Gemmatimonadota were not significantly greater, and the relative abundances of Chloroflexota and Acidobacteriota were significantly greater in the T group than in the Ctrl group.

At the genus level, 1154 taxa in the Ctrl and T groups were classified. The top 30 genera in each group are presented in Fig. 2B. The dominant genera in both groups were *Sphingomonas* (31.4% vs 41.0%), *Nocardioides* (11.9% vs 8.9%), and *Rhodanobacter* (5.2% vs 2.3%). The Metastats method was used to determine the significant (*P* < 0.05) differences in the relative abundances of the dominant genera between the two groups. The relative abundance of *Sphingomonas* was not significantly greater in the T group than in the Ctrl group, whereas the relative abundances of *Nocardioides* and *Rhodanobacter* were not significantly lower. Partial least squares discriminant analysis (PLS­DA) revealed variation in the microbial communities between the T and Ctrl samples. At the genus level, the T samples were significantly separated from the Ctrl samples (Fig. 2C). Next, statistical analysis of taxonomic and functional profiles (STAMP) analysis was used to compare the top 10 most abundant genera (false discovery rate [FDR] < 0.05 indicated statistical significance) (Fig. 2D). The relative abundance of *Nocardioides* was significantly lower in the T group than in the Ctrl group. *Phycicoccus* and *Leifsonia* also significantly decreased, whereas *Sphingomonas* and *Pseudolabrys* significantly increased. In both the T and Ctrl soil samples, *Sphingomonas*, *Nocardioides*, *Phycicoccus*, and *Pseudolabrys* were the dominant genera.

These changes in relative microbial abundances seem to be associated with DX­9 inoculation in the soil and may thus be influenced by DX­9. DX­9 inoculation not only altered the dominant phyla in the soil but also affected the genera.

### Correlation analysis between microbes and soil properties

**Fig. 3:**
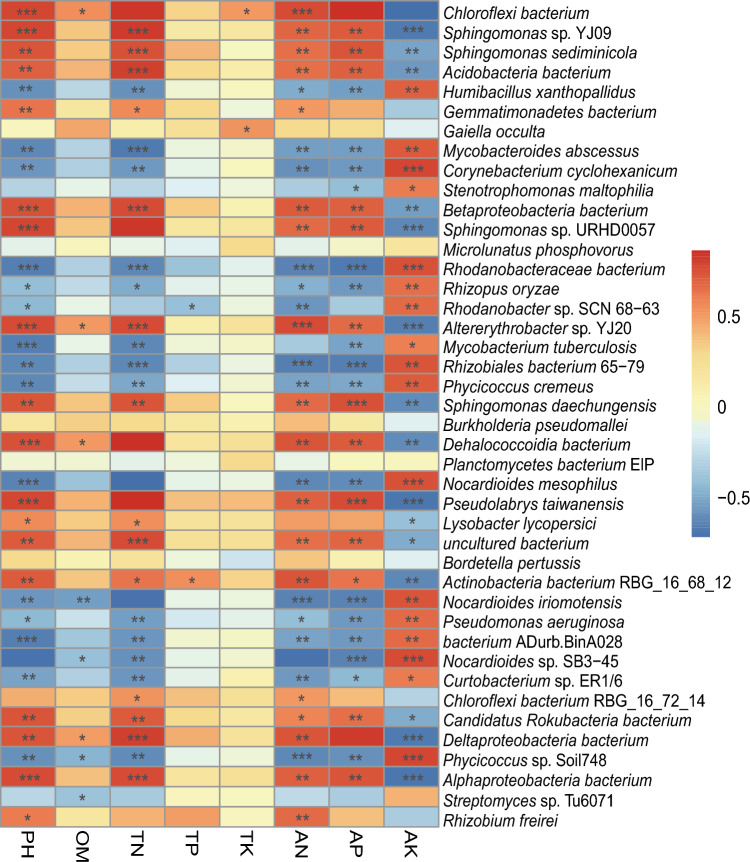
Heatmap of Spearman’s rank correlation coefficients (*r* values between − 1 and 1) between relative microbial abundances and soil properties. *r* < 0 indicates a negative correlation, and *r* > 0 indicates a positive correlation. The blue to red gradient indicates low to high abundance. *P < 0.05, **P < 0.01, ***P < 0.001, indicating statistically significant correlation between relative microbial abundance and soil properties

The composition of soil microbial populations was strongly correlated with soil chemical properties. The chemical properties of the bulk soil samples used in this study are shown in Table 1. DX­9 inoculation significantly increased TN, TP, AN, and AP. Spearman correlation analysis was used to assess the correlations (based on the r value and *P* < 0.05) between the soil properties and the relative abundances of the top 30 microbial groups (Fig. 3). The relative abundances of microbes were not significantly correlated with TP or TK. The relative abundances of many microbes, including *Sphingomonas*, *Altererythrobacter*, and *Pseudolabrys*, were positively correlated with pH, OM, TN, AN, and AP and negatively correlated with AK. The relative abundances of microbes such as *Nocardioides*, *Rhodanobacter*, *Streptomyces*, and *Ferruginibacter* were positively correlated with AK. The relative abundances of microbes such as *Phycicoccus* sp. and *Altererythrobacter,* were positively correlated with AK and negatively correlated with pH, OM, TN, AN, and AP.

These results indicated that correlations exist between soil properties and relative microbial abundances, which were affected by DX­9 inoculation.

### Effects of DX-9 inoculation on soil metabolites

**Fig. 4:**
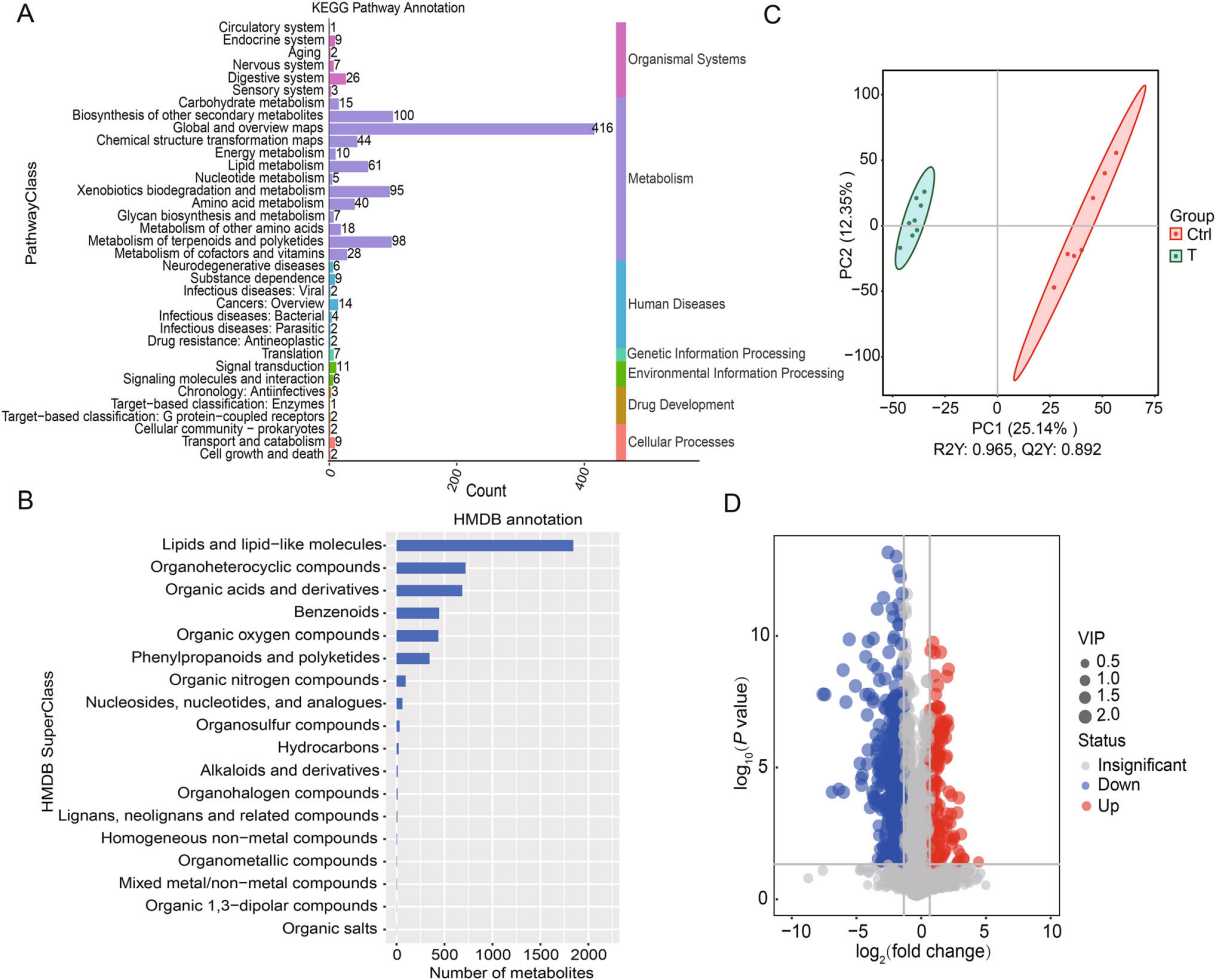
Metabolite annotation and analyses. **A** KEGG database-based metabolite annotation. **B** HMDB database-based metabolite annotation. **C** Metabolite partial least squares discriminant analysis (PLS­DA). **D** Differentially abundant metabolites between the T and Ctrl groups in a volcano plot. The *x* axis shows log_2_ (fold change), and the y axis shows − log_10_ (*P* value). The red, blue, and gray dots represent upregulated, downregulated, and nonsignificant metabolites, respectively. Significant differences were determined on the basis of − log_10_ (*P* value), *P* < 0.05

To assess the effects of DX­9 inoculation on soil functions, we analyzed the tuber rhizosphere metabolome. Liquid chromatography-tandem mass spectrometry (LC­MS/MS) detected 7020 metabolites. Metabolite annotation was performed using the Kyoto Encyclopedia of Genes and Genomes (KEGG) database and Human Metabolome Database (HMDB) to help understand the biological functions of the metabolites. The KEGG analysis divided the 1086 annotated metabolites into 7 primary categories and 35 subcategories (Fig. 4A; Table S1). The most abundant category was metabolism, and the top three most abundant subcategories were global and overview maps, biosynthesis of other secondary metabolites, and metabolism of terpenoids and polyketides. The HMDB analysis divided the 4,733 annotated metabolites into 18 classes. The three most abundant classes were lipids and lipid-like molecules, organoheterocyclic compounds, and organic acids and derivatives (Fig. 4B).

PLS­DA revealed a significant separation between the Ctrl and T groups. This result indicated that the relative abundance of soil metabolites changed after DX­9 inoculation (Fig. 4C). The volcano plot revealed 750 significantly differentially abundant metabolites in the T group compared with the Ctrl group (188 upregulated and 562 downregulated) (Fig. 4D). We were interested in the metabolites upregulated by DX­9, as we speculated that they may well suppress the CS pathogen in the soil. The top 10 differentially upregulated metabolites (based on the fold change in their abundance) are shown in Fig. S3. The top three metabolites were phytolaccoside A (27.36-fold increase in abundance), 7,8­dihydropteroic acid (12.63-fold increase in abundance), and novobiocin (12.50-fold increase in abundance). The antioxidant azafrin (7.38-fold increase in abundance) also significantly increased in the T group.

**Fig. 5:**
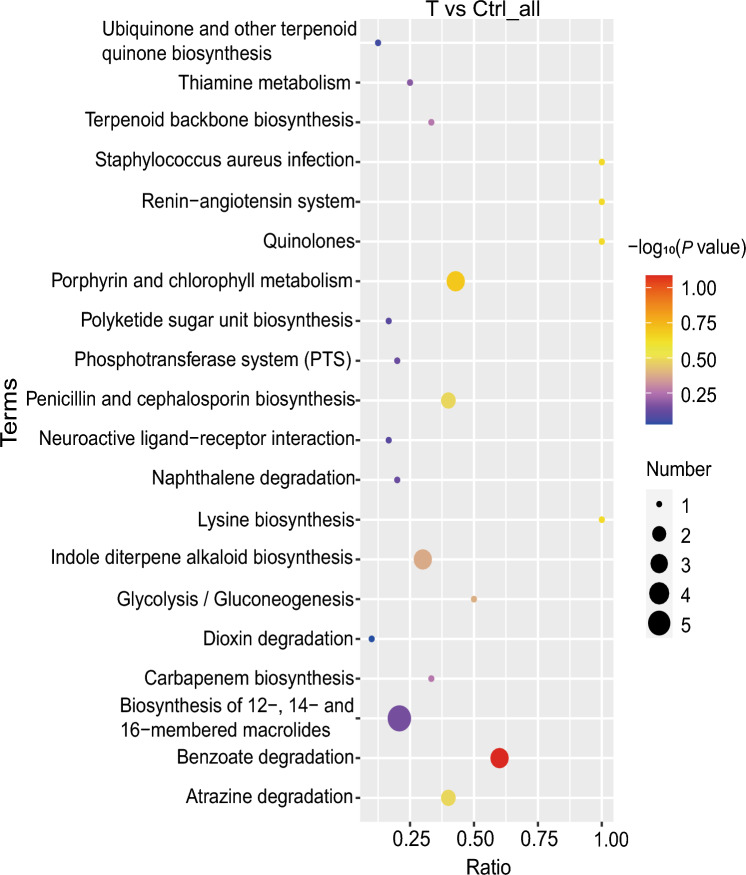
KEGG analysis of differentially abundant metabolite pathways between the T and Ctrl groups. The *x* axis shows the enrichment ratio (number of differentially abundant metabolites in the metabolic pathway/total number of metabolites identified in this pathway), and the *y* axis shows the pathway names. The color of the dot represents − log_10_ (*P* value), and the size of the dot represents the number of differentially abundant metabolites

These significantly differentially abundant metabolites were subjected to KEGG analysis; in Fig. 5 we highlight the enrichment ratios and *P* values. The major pathways were benzoate degradation (*P*: 0.08, R: 0.60), porphyrin and chlorophyll metabolism (*P*: 0.20, R: 0.43), penicillin and cephalosporin biosynthesis (*P*: 0.33, R: 0.40), atrazine degradation (*P*: 0.33, R: 0.40), indole diterpene alkaloid biosynthesis (*P*: 0.42, R: 0.3), and the biosynthesis of 12­, 14­, and 16­membered macrolides (*P*: 0.71, R: 0.21), which are all related to metabolism (Table S1). Thus, DX­9 inoculation significantly affected these metabolite pathways.

Taken together, these findings indicated that the metabolome of DX­9-inoculated tuber rhizosphere soil exhibited changes in many metabolic pathways compared with the metabolome of the uninoculated control.

## Discussion

Most potato-growing areas have widespread CS disease, which is difficult to control. In conventional strategies, chemical agents, including soil fumigation agents, are used to control CS. However, these strategies are not only expensive but also increase soil pollution and are inefficient (Dees and Wanner 2012). Biological control is an attractive alternative for controlling CS. Microbial inoculants are beneficial microbial agents that can improve soil health, increase nutrient availability, promote plant growth, and control pathogens (Trabelsi and Mhamdi 2013). In this study, we confirmed that soil inoculation with *B. atrophaeus* DX­9, as a biocontrol agent, decreased the CS. We used an integrated metabolomic and metagenomic analysis to assess the effects on the microbial communities and soil metabolites to understand the associations between DX­9 inoculation and the observed CS control.

### DX­9 inoculation effectively controls CS

The overuse or inappropriate use of chemical agents, environmental pollution and food safety issues are serious problems, so biocontrol measures are important alternatives in sustainable agriculture (Dai 2012). The use of beneficial microbes as biocontrol agents to manage CS has long been known to be effective. Nonpathogenic *Streptomyces diastatochromogenes* PonSSII and *Streptomyces albogriseolus* inhibited *S. scabiei* by using antibiotic analogs (Eckwall and Schottel 1997). *Streptomyces melanosporofaciens* EF­76 significantly decreased the incidence of CS (Beauséjour et al. 2003). *Streptomyces* A1RT effectively suppressed multiple CS pathogens (Sarwar et al. 2018). *Streptomyces* sp. WoRs-501 decreased pathogen populations in soil and strongly suppressed CS (Kobayashi et al. 2015). Moreover, *Pseudomonas* sp. LBUM 223 limited CS pathogen growth to control CS in potato (Renée et al. 2015). *Brevibacillus laterosporus* AMCC100017 decreased CS severity, with a biocontrol efficacy of 70.5% (Chen et al. 2017). Furthermore, *Bacillus* spp. constitutes a large group of beneficial microbes that can be used to control CS. *Bacillus* sp. sunhua suppressed *S. scabiei* in soil and decreased the infection rate from 75 to 35% (Han et al. 2005). *B. amyloliquefaciens* BAC03 significantly reduced CS severity (Meng et al. 2013) *B. subtilis* znjdf1 (combined with *Trichoderma harzianum* znlkhc1) decreased the CS disease index and increased the potato yield (Wang et al. 2019). These studies discussed the effects on CS control in terms of physiological and biochemical mechanisms but did not include omics analyses.

In our study, an antagonistic bacterial isolate (DX­9) was identified as *B. atrophaeus*. We evaluated its effect against CS in a pot assay and established that it significantly decreased the CS disease rate from 97.2% in the Ctrl group to 64.3% in the T group and had high control efficacy (Fig. 1A–E). The results of our field trial were consistent with those of the pot assay (Fig. 1F, G), in that DX-9 also decreased the number of CS pathogens (on the basis of relative level of *txtA*) in the soil (Fig. 1E, G). These findings demonstrated that *B. atrophaeus* DX­9, which is a *Bacillus* spp., is a potential inoculant for controlling CS that may directly suppress the CS pathogen population. To our knowledge, this is the first report on the use of *B. atrophaeus* to control a CS pathogen and the first report on the use of multi-omics approaches to study the mechanisms underlying CS control by a *Bacillus* isolate.

### DX­9 inoculation improves soil properties

Soil microbes are among the most important components for enhancing soil quality, as they can alter soil nutrient availability. To assess the effects on soil nutrients, we assessed the changes in soil chemical properties after DX-9 inoculation (Table 1). Here we observed that DX-9 significantly increased TN, TP, AN, and AP. Symbiotic N fixation in soil occurs via bacteria (which exclusively fix N as they express the key enzyme nitrogenase) living in plants (Stein and Klotz 2016). Many soil bacteria can fix N, including *Bacillus* spp. (Saharan and Nehra 2011). Thus, we hypothesized that *B. atrophaeus* DX­9 could fix N to increase TN and AN. Moreover, DX­9 inoculation affected the relative abundance of various resident soil microbes (Fig. 2A, B). The relative abundance in genera that can fix N, including *Rhizobium*, *Bradyrhizobium*, and *Agrobacterium,* increased (Table S2).

P is an essential macronutrient, but is often insoluble in soil and therefore unavailable to plants. Thus, soil P levels have to be supplemented by addition of chemical P fertilizers. Overuse of chemical P leads to many problems related to soil health, and both freshwater and marine resources (Tilman et al. 2001). In our study, TP was significantly increased after DX-9 inoculation. We hypothesized that DX-9 may have helped to prevent P loss from the soil after a potato growth period, as the application of microbial agents can improve soil structure, including increasing soil aggregates (Deng et al. 2015). AP was also significantly increased after DX-9 inoculation. Microbial inoculants (including *Pseudomonas, Azospirillum, Azotobacter, Bacillus*, and mycorrhizae) can increase soil P availability by solubilizing and mobilizing P, via the production of organic acids and the enzymes phosphatase and phytase (Calvo et al. 2014). The relative abundances of various microbes that can increase P availability (including *Enterobacter*, *Salmonella*, *Flavobacterium*, *Rhizophagus*, and *Aspergillus*) was significantly increased in our study (Table S2). Consistent with the increased AP in the current study, our previous study revealed that DX-9 can produce organic acids to increase AP (Cao et al. 2023a, b). In the current study, the metabolomic analysis revealed that DX­9 inoculation increased organic acids, such as 2­hydroxyacetaminophen sulfate, which is an organic sulfuric acid derivative. The third most abundant HMDB class was organic acids and their derivatives (Fig. 4B).

DX­9 inoculation did not significantly affect pH, OM, TK, or AK (Table 1). Although the Spearman correlation analysis revealed that many microbes were positively correlated with increased AK (Fig. 3), they did not increase the soil K content after DX-9 inoculation (TK was unchanged, and AK was not significantly decreased). We hypothesized that DX-9 inoculation altered the abundance of various microbes, but the abundance of microbes positively correlated with AK was lower than the abundance of microbes negatively correlated with AK, and the latter set of microbes offset the former. These results showed that DX­9 inoculation changed the soil properties, but further study of the molecular mechanisms is needed to validate the functional genes of DX­9.

### DX­9 inoculation alters the microbial community structure

A microbial inoculant introduced to soil may disturb the balance of the soil microbial community and change its structure (Babalola 2014). However, the alpha diversity indices of the soil microbes (species richness and diversity indices) indicated that there were no significant differences between the Ctrl and T groups (Table 2). Plant root exudates can benefit rhizosphere microbes, increasing certain plant growth-promoting microbes and, thereby, decreasing microbial diversity (Jacoby and Kopriva 2019; Kuzyakov and Blagodatskaya 2015). Additionally, excessive application of organic or chemical fertilizer can lead to changes in microbial diversity and community structure (Gupta et al. 2022). In our study area, fertilizer was overused compared with other potato-growing areas in China (Jia et al. 2018), and the amount of nutrients needed for potato growth may well have been exceeded. We speculate that this is one reason why the microbial alpha diversity in our study was not affected by DX­9 inoculation.

In our study, taxonomic annotation revealed that there were differences in the microbial communities between the Ctrl and T groups. At the phylum level, Actinomycetota, Pseudomonadota, and Chloroflexota were dominant. The relative abundance of Actinomycetota decreased in the T group compared with the Ctrl group, whereas the relative abundances of Pseudomonadota and Chloroflexota increased (Fig. 2A). Pseudomonadota can fix symbiotic nitrogen and biodegrade polycyclic aromatic hydrocarbons (Debruyn et al. 2009). Chloroflexota exhibits metabolic diversity and adaptability, so it is useful for water treatment, pollution degradation, and energy production (Freches and Fradinho 2024).

At the genus level, *Sphingomonas*, *Nocardioides*, and *Rhodanobacter* were dominant. The relative abundance of *Sphingomonas* not significantly increase in the T group compared with the Ctrl group, and the relative abundance of *Nocardioides* and *Rhodanobacter* also not undergo a significant decrease (Fig. 2B). Furthermore, the PLS­DA and STAMP analyses indicated that there were different microbial community characteristics between the T and Ctrl groups (Fig. 2C, D). STAMP analysis also revealed that *Sphingomonas* significantly increased in the T group compared with the Ctrl group and that *Nocardioides* significantly decreased (Fig. 2D).

*Sphingomonas* is an important microbial genus that can degrade a variety of aromatic compounds and pollutants, promote plant growth, and alleviate abiotic stresses (Liu et al. 2017). *Sphingomonas* sp. Hbc­6 has been reported to improve plant growth and drought tolerance, and it is a potential agricultural biofertilizer. *Nocardioides* can modulate soil C transformation and may play a role in soil bioremediation and detoxification in contaminated regions (Miao et al. 2020). The reduced relative abundance of *Nocardioides* may explain the absence of any significant decrease in OM. *Rhodanobacter* spp. are known to cause N loss and plant diseases (Kumar et al. 2014). Therefore, a reduced relative abundance of *Rhodanobacter* is beneficial for plant health.

The relative abundances of certain disease control-related bacteria were also changed (Fig. 2B). In particular, *Lysobacter* and *Methylobacterium* increased after DX-9 inoculation, indicating that DX-9 affected the abundance of certain antagonistic bacteria. *Lysobacter* can produce a large number of extracellular hydrolytic enzymes and active secondary metabolites, which have strong antagonistic activity against pathogenic bacteria, fungi, oomycetes and so on. *Lysobacter* has become an important biocontrol bacterium and a source of natural bioactive products (Xie et al. 2012; Yue et al. 2022). Finally, *Methylobacterium asv41* can inhibit spore germination and mycelial growth (Li et al. 2022).

Based on our findings, DX-9 inoculation has a significant effect on soil microbial community structure and soil properties (especially N and P). There were complex correlations among the inoculant, microbial community, and soil. On the one hand, DX-9 significantly increased TN, TP, AN, and AP, via bacteria, potentially as a result of N fixation and the control of P loss. The relative abundances of these bacteria increased and reflect the fact that the soil microbial community structure had been changed by DX-9. These changes may have affected the soil N and P cycles and how effectively N and P could be utilized, thereby improving the soil properties. On the other hand, the improved soil properties also affected the microbial community structure. The relative abundances of certain disease control-related bacteria, such as *Lysobacter* and *Methylobacterium*,were increased (Fig. 2B). These improved soil properties enhanced plant growth, and the change in microbial community structure, soil properties and plant growth, combined with the antagonistic effect of DX-9 on the pathogen, reduced the incidence of CS.

### DX­9 inoculation alters soil metabolites

Microbial inoculants can effectively control pathogens by altering soil metabolites. KEGG annotation (Fig. 4A) revealed that metabolism was the most enriched level-1 pathway (937 of 1086 annotated metabolites). Global and overview maps (416 of 1086) and biosynthesis of other secondary metabolites (100 of 1086) were the most enriched level-2 pathways. The biosynthesis of antibiotics (121 of 1086) and metabolic pathways (119 of 1086) were the most enriched level-3 pathways. Many effective biocontrol compounds are antibiotics or secondary metabolites (such as lipopeptides, geldanamycin, phenazine compounds, and antimicrobial peptides). HMDB analysis revealed that the most abundant soil metabolites were lipids and lipid-like molecules (Fig. 4B), and a high abundance of lipopeptides may play an important role in CS control by DX­9.

KEGG analysis of the many significantly differentially abundant metabolites in the T versus Ctrl group (Fig. 5) indicated that the significantly enriched pathways were related to metabolism, such as xenobiotic biodegradation and metabolism, biosynthesis of other secondary metabolites, and metabolism of cofactors and vitamins (Table S1; Fig. 5). The levels of phytolaccoside A, 7,8­dihydropteroic acid, novobiocin, and azafrin were significantly greater in the T group than in the Ctrl group (Fig. S3). Phytolaccoside A is a triterpenoid saponin that has anti-inflammatory, antifungal, and anticancer activities (Bailly and Vergoten 2020). 7,8-Dihydroneopterin has antioxidant activity and scavenges oxidants such as hypochlorite (Ghodsian et al. 2022). Novobiocin is an antibiotic that inhibits DNA gyrase by binding to the ATP-binding site in the ATPase subunit. Azafrin exhibits antioxidant activity (May et al. 2017). These metabolites may control CS through their antagonistic effects on CS pathogens. However, further studies are needed for verification.

Microbes are always correlated with soil metabolites, as they produce a wide range of metabolites, including enzymes, indole acetic acid, and antibiotics. We analyzed the changes in the relative abundances of microbes in combination with changes in metabolic pathways in the soil. The relative abundance of *Sphingomonas* (which degrades C sources such as phenol and other aromatic hydrocarbons in processes involving secondary metabolites (Gong et al. 2016)) significantly increased after DX­9 inoculation (Figs. 2D, 3), improving soil ecosystems. KEGG analysis of the soil metabolites revealed that the most enriched primary category after DX­9 inoculation was metabolism (Fig. 4A). However, we did not find a correlation between changes in other beneficial microbes and changes in metabolites. For example, changes in the abundances of *Streptomyces*, *Bacillus*, and *Pseudomonas* were correlated with the levels of antibiotics, phenazine compounds, or peptides but were not correlated with soil properties (Fig. 3). More work is needed to study the correlation between microbes and metabolites. We hypothesized that DX­9 inoculation of the soil changes the soil properties to promote plant metabolite secretion to control CS pathogens. Thus, DX­9 in soil is hypothesized to directly alter the microbial community and indirectly alter soil metabolites (by promoting plant metabolite secretion), suppressing the number of CS pathogens. Further studies will focus on determining whether the mechanisms by which DX-9 controls CS are direct or indirect. We will carry out experiments to confirm the inhibitory function of specific metabolites and the CS-control mechanisms of DX­9.

In this study, we identified *B. atrophaeus* DX­9, which significantly decreased the CS disease rate and disease index in potatoes. DX­9 inhibited the number of CS pathogens in the soil and improved soil properties such as AN and AP. An integrated metagenomic and metabolomic analysis elucidated the changes in the microbial communities and metabolites in the soil and thereby helped to explain the effects of DX­9 inoculation in the soil. DX­9 inoculation increased the relative abundance of microbes that can improve soil properties and plant disease tolerance. Metabolomic analysis revealed that the soil metabolites were affected by changes in the microbial community, which were influenced by DX­9 inoculation. Our findings revealed that DX­9 inoculation controlled CS by altering the soil properties, soil microbial community, and soil metabolites. Hence, DX­9 is a potential microbial inoculant for controlling CS and provides a basis for further research on microbial inoculants for controlling soilborne diseases.

## Materials and methods

### Isolation and selection of antagonistic bacteria

The highly pathogenic *S. scabiei* strain 4.1765 was obtained from the China General Microbiological Culture Collection (CGMCC no. 4.1765), cultured on oatmeal medium agar (OMA; Coolaber Co., Ltd, Beijing, China) for 5 days, and then cultured in tryptic soy broth (TSB) liquid medium (Becton, Dickinson and Company, Sparks, MD, USA) at 28 °C in a rotary shaker (200 rpm) for 3 days. The culture suspension was then evenly spread on potato dextrose agar (PDA) media (Becton, Dickinson and Company, Sparks, MD, USA) plates for antagonistic bacterial screening.

Bacteria were isolated from rhizosphere soil collected from a potato-growing field in Dingxi city, Gansu Province China, based on a previously described method (Yilmaz et al. 2006). Briefly, 1 g soil was suspended in 10 mL sterile water, vortexed for 1 min, placed in a 60 °C water bath for 1 h, serially diluted from 10^–1^ to 10^–6^, plated on nutrient agar (NA) media, and incubated at 37 °C for 48 h. Bacterial isolates were picked, purified, and stored on NA medium at 4 °C. The antagonistic bacteria were then subjected to screening using a dual-culture method with slight modifications. Briefly, the bacterial isolates were incubated at 37 °C in a rotary shaker (200 rpm) in Luria Bertani (LB) liquid media for 3 days and then mixed with sterile water to create bacterial suspensions of the same concentration (*OD*_600_ = 1). Next, 6 μL of each bacterial suspension was spotted in the center of a PDA plate spread with *S. scabiei* 4.1765 and incubated at 28 °C for 3 days. Bacteria that inhibited *S. scabiei* growth were considered antagonistic strains.

The strain that caused the largest inhibition zone diameter was identified by 16S rDNA sequencing and used for further study. Briefly, the genomic DNA of the selected strain was extracted using a bacterial genomic DNA kit (TIANGEN, Beijing, China) and stored at 4 °C. The 16S rDNA was amplified using the bacterial universal primers 27F (5′-AGAGTTTGATCCTGGCTCAG-3′) and 1492R (5′-GGTTACCTTGTTACGACTT-3′) by polymerase chain reaction (PCR) (Wu et al. 2016). DNA sequence homology was analyzed using the BLAST tool from the NCBI (http://blast.ncbi.nlm.nih.gov/Blast.cgi). On the basis of sequence alignment, a phylogenetic tree of this strain and other Bacillus strains was constructed using the neighbor-joining method in MEGA (v 7.0.21).

The bacterial strain was serially diluted (1 × 10^–7^ to 1 × 10^–10^ cfu/mL) and the concentration for use in subsequent experiments was determined on the basis of the inhibition zone diameter using the above screening method.

### Pot assay

*S. scabiei* 4.1765 was prepared and used to inoculate plants according to our previous methods (Zhao et al. 2022). The selected antagonistic strain was incubated in LB liquid medium at 37 °C in a rotary shaker (200 rpm) for 5 days, harvested by centrifugation (5,000×*g* for 10 min), washed with sterile water, resuspended in sterile water, and serially diluted to 1 × 10^9^ cfu/mL for inoculation.

*Solanum tuberosum* L. cv. Favorita plants (a CS-susceptible potato cultivar) were prepared as previously described (Zhao et al. 2022). Briefly, potato seedlings were cut into single‐node explants of similar length and cultured on solid Murashige and Skoog (M&S) media (pH 5.8) at 24 °C with a 16/8 h light/dark photoperiod. After 2 weeks, seedlings of similar size were transferred to 25-cm-diameter pots (five seedlings per pot) filled with the same amount of sterilized vermiculite. Then, 10 mL inoculation buffer was poured near the roots of seedlings. Therefore, a total of 50 mL inoculation buffer was inoculated into each pot. The plants were grown in a greenhouse under the same conditions (24 °C, 16/8 h light/dark photoperiod). The pots were divided into the following three groups: (1) a negative control (NC) group (*S. scabiei*-uninoculated plants); (2) control (Ctrl) group (*S. scabiei*-inoculated plants); and (3) treatment (T) group (plants co-inoculated with *S. scabiei* and the antagonistic strain). There were 3 pots to replicate each treatment. At 90 days after the seedlings were planted, the tubers were harvested and assessed for CS.

### Field trial

A field trial with a randomized block design was set up in Huidong County (114° 885 E, 22° 710 N), Huizhou city, Guangdong Province, China. It has loamy clay acidic soil and a subtropical marine monsoon climate, with a mean annual temperature of 21.7 °C, a mean annual sunshine duration of 2350 h, a mean annual precipitation of 1805 mm, and a mean relative humidity of 78%, pH 5.36. Potatoes have been continuously grown at this site for 5 years, and plantings had a high percentage of CS in the past year (more than 90%) according to our field survey.

The site was divided into two groups: (1) the control (Ctrl), which was not inoculated with the antagonistic strain, and (2) the treatment (T), which was inoculated with the antagonistic strain. Each group included 400 m^2^ (20 × 20 m^2^) of land, with five rows, a 90-cm ridge distance, and 25 cm plant spacing. The two groups experienced the same conditions during the course of the experiment (temperature, sunshine duration, precipitation, soil properties, and agricultural management).

*Solanum tuberosum* L. cv. Favorita was the cultivar used in this filed study. The YaraMila compound fertilizer of potassium sulfate (N–P_2_O_5_–K_2_O = 12–11–18) was applied at 2200 kg/ha^−2^. Seeds in the T group were soaked in the antagonistic strain inoculum for 15 min and in Ctrl were soaked in the water inoculum for the same time. The seeds in both groups were sown in the soil on December 20, 2022 and the soil was then covered with a plastic film for 3 weeks. Potatoes were harvested on April 20, 2023. For CS severity assessment, potato collected from eight random points (five potato plants per point in a row) with an “S” shape in each group. So eight replicates points per group were selected to collect potatoes. CS severity was assessed one week after harvest. Bulk soil and rhizosphere soil samples were also collected.

### Disease assessment

Tubers > 2 g were selected and washed under running water, and CS severity was evaluated as previously described (Zhao et al. 2022).

The disease index was calculated to assess the CS severity using the following equation:

1 $${\text{Disease index }} = { }\left[ {\sum { }\left( {{\text{n}}_{1} \times { }1{ } + {\text{ n}}_{2} \times { }2{ } + {\text{ n}}_{3} \times { }3{ } + {\text{ n}}_{4} \times { }4{ } + {\text{ n}}_{5} \times { }5} \right)/{ }\left( {{\text{N }} \times { }5} \right)} \right]{ } \times { }100$$

where n_1_ to n_5_ are the numbers of tubers with each of the following scores (based on the percentages of tubers covered by lesions): 0 (no lesions), 1 (0–12.5%), 2 (12.6–25%), 3 (26–50%), 4 (51–75%), and 5 (76–100%), respectively, and N is the total number of tubers assessed.

The control efficacy was calculated using the following equation:

2 $${\text{Control efficacy}} = { }\left( {{\text{disease index of Ctrl group }} - {\text{ disease index of T group}}} \right)/{\text{disease index of Ctrl }} \times { }100{\text{\% }}$$

The disease rate was calculated using the following equation:

3 $${\text{Disease rate}} = \left( {{\text{number of infected tubers}}/{\text{total number of tubers}}} \right){ } \times { }100{\text{\% }}$$

The groups were compared using Student’s *t* test (unpaired, two-tailed) in GraphPad Prism (v9.3.0).

### Soil sampling and preparation

The soil samples were collected from the field during potato harvesting according to previous methods with slight modifications (Cao et al. 2023a, b). Briefly, rhizosphere soil samples and bulk soil samples were obtained from eight random points (five potato plants per point), in an “S” shape, in each field trial group. The rhizosphere soil samples comprised soil tightly adhering to all the tubers of one potato plant. The five samples from each point were pooled together to generate one composite sample, so eight composite samples per group were collected. The bulk soil samples comprised soil collected from the region around tubers in one potato plant (at a depth of 20 cm), and the five samples from each point were pooled together, as was the case for one bulk soil sample. Eight composite bulk samples were also collected.

The bulk soil samples were stored at 4 °C before measuring the following soil properties were measured: pH, OM, TN, TP, TK, AN, AP, and AK. The soil properties were measured using previously methods (Wilke 2005). The rhizosphere soil samples were stored at − 80 °C for subsequent qRT-PCR and omics analyses.

### QRT-PCR quantification of CS pathogen DNA in soil

To assess the number of CS pathogens in rhizosphere soil samples, qRT-PCR was used to quantify the relative level of *txtA* gene (genes for thaxtomin A biosynthesis), as previously described (Manome et al. 2008). This gene elicits CS symptoms and is located on a conserved and mobile pathogenicity island in pathogenic *Streptomyces* spp. Given the strong association between pathogenicity and thaxtomin the production involving the *txtA* gene, *txtA* can serve as a marker for pathogenic *Streptomyces* spp. (Braun et al. 2017). Briefly, soil DNA was extracted using a HiPure Soil DNA Kit (Magen, Guangzhou, China). The online Primer-BLAST tool from NCBI (https://www.ncbi.nlm.nih.gov/tools/primer-blast/) was used to design primers based on the *S. scabiei txtA* gene (GenBank: FN554889.1) for amplification on a CFX96 Touch qRT-PCR Detection System (Bio­Rad, Hercules, CA, USA). The sequence of the forward primer was 5′­CACGTACGCGCAGTTCAATG­3′, and the reverse primer was 5′­AGATGATGTAGGCGGGAC­3′ (Wanner 2007). There were five technical replicates and three biological replicates. The relative level of *txtA* gene was calculated using the 2^−∆∆CT^ method (Livak and Schmittgen 2001). 16S rRNA was used as an internal control (Sagova et al. 2017).

### Metagenomics analysis

Total DNA was extracted from the rhizosphere soil samples using a TIANamp Soil DNA Kit (TIANGEN Biotech Co., Ltd., Beijing, China), and the DNA concentration was measured using a Qubit^®^ dsDNA Assay Kit on a Qubit^®^ 2.0 Fluorometer (Life Technologies, CA, USA). Library construction and high-throughput sequencing were conducted by TIANGEN Biotech (Beijing) Co., Ltd. (Beijing, China; https://www.tiangen.com/; accessed on January 1, 2024). An Illumina HiSeq PE150 platform was used to generate the raw sequencing data. After the overlapping reads and low-quality reads were removed, the clean data were assembled and analyzed using MEGAHIT (v1.2.9).

ORF prediction was performed using MetaGeneMark (v3.38; http://topaz.gatech.edu/GeneMark/), and an initial nonredundant gene catalog was constructed using CD-HIT (v4.8.1; http://www.bioinformatics.org/cd-hit/). The clean data were aligned to this catalog using Bowtie2 (Bowtie2.2.6), which calculates the number of aligned reads of each gene in each sample. The abundance of each gene was calculated basis of the number of aligned reads and the gene length. After the final nonredundant gene catalog (unigenes) was obtained, the basic statistics of the catalog were calculated, and correlation analysis, was conducted. Alpha-diversity indices were calculated using QIIME II (v1.9.1). Unigene sequence alignment with the NCBI NR database (v2018­01­02; https://www.ncbi.nlm.nih.gov/) was then performed using DIAMOND (v0.9.22). The sequences underwent taxonomic annotation by using the lowest common ancestor (LCA) algorithm (top alignment percentage = 10% [default], i.e., the%. top 10% of the best hits) based on DIAMOND (Wood and Salzberg 2014).

Using the gene abundance results and LCA taxonomic annotation, the abundances at each taxonomic level in each sample and corresponding gene abundance tables were acquired, and a species distribution histogram was generated. To reveal the variation in the microbial communities among soil samples, partial least squares discriminant analysis (PLS­DA) was performed. To identify differences in taxa abundances between groups, statistical analysis of taxonomic and functional profiles (STAMP) analysis was used.

### Metabolomics analysis

The Ctrl and T rhizosphere soil samples were subjected to metabolomics analysis by TIANGEN Biotech (Beijing) Co., Ltd. (Beijing, China; https://www.tiangen.com/; accessed on January 1, 2024).

The soil metabolites were extracted according to previously described methods with modifications (Swenson and Northen 2019). Briefly, 1 g soil sample was mixed with 400 mL extraction solution (methanol:acetonitrile:water, 2:2:1), frozen in liquid nitrogen for 1 min, thawed, vortexed for 30 s, and ultrasonicated in ice water for 10 min. These steps were repeated three times. Next, the samples were incubated at 40 °C for 1 h and then centrifuged (12,000 rpm for 15 min) to obtain the supernatants.

LC­MS/MS was used for the metabolomics analysis. The compounds in the supernatants were separated using a Vanquish Flex Ultra-Performance Liquid Chromatography (UPLC) system (Thermo Fisher Scientific, Waltham, MA, USA) with a Waters ACQUITY UPLC BEH Amide column (2.1 mm × 100 mm, 1.7 μm; Waters, Milford, MA, USA). Phase A was a water solution containing 25 mmol/L ammonium acetate and 25 mmol/L ammonia; phase B was 80 vol% acetonitrile solution. The chromatographic gradient program, with a flow rate of 0.4 mL/min, was as follows: 0–0.25 min, 2% B; 0.25–10 min, 2–98% B; 10–13 min, 98% B; 13–13.1 min, 98–2% B; and 13.1–15 min, 2% B. Mass spectrometry was performed using a Thermo Q Exactive HFX system (Thermo Fisher Scientific, Waltham, MA, USA) with the following conditions: a sheath gas flow rate of 30 Arb, an aux gas flow rate of 25 Arb, a capillary temperature of 350 °C, a full-MS resolution of 60,000, an MS/MS resolution of 7500, collision energy of 10/30/60 in normalized collision energy (NCE) mode, and a spray voltage of 3.6 kV (positive) or − 3.2 kV (negative).

The raw metabolomics data were preprocessed using Proteowizard (v3.0.9), and peak extraction and peak alignment were performed using the XCMS package (v1.42.0) in R (v3.3.2). Metabolite identification was performed in R (v3.3.2) using the mzCloud database (https://www.mzcloud.org/) and the MassBank database (http://www.massbank.jp/). Metabolite annotation was performed using the KEGG database (http://www.genome.jp/kegg/) and HMDB (https://hmdb.ca/metabolites). PLS­DA was performed using the Ropls package in R (v3.3.2). The significantly differentially abundant metabolites, based on variable importance in projection (VIP, which reflects a metabolite’s contribution to group separation) > 1, fold change > 2 or < 0.5, and *P* < 0.05, were determined using the Wilcox.test/t.test functions in R (v3.3.2). KEGG enrichment analysis (*P* < 0.05 indicated significant enrichment) of the significantly differentially abundant metabolites between the T and Ctrl groups was conducted in R (v3.3.2).

## Supplementary Information

Below is the link to the electronic supplementary material.

Supplementary file 1 (DOCX 1803 KB)

## Data Availability

The clean data were deposited in the Science Data Bank (10.57760/sciencedb.09118; deposited on May 29, 2024).
