# Supplementary material for: Bacillus atrophaeus DX-9 biocontrol against potato common scab involves significant changes in the soil microbiome and metabolome
Source: aBIOTECH. 2025 Feb 18;6(1):33–49. doi: 10.1007/s42994-025-00199-3 (PMC11889282; doi:10.1007/s42994-025-00199-3)
Supplement: Supplementary file 1 — (DOCX 1803 KB) [file 42994_2025_199_MOESM1_ESM.docx]

Bacillus atrophaeus DX-9 biocontrol against potato common scab involves significant changes in the soil microbiome and metabolome

**Jingjing Cao ^1, 2^, Yue Ma^1, 2, 3^, Jing Fu^4^, Zhiqin Wang ^1, 2^, Yonglong Zhao ^1, 2^, Naiqin Zhong^1, 2, 3, 5^, and Pan Zhao ^1, 2, 5, *^**

^1^State Key Laboratory of Plant Genomics, Institute of Microbiology, Chinese Academy of Sciences, Beijing, 100101, China

^2^Engineering Laboratory for Advanced Microbial Technology of Agriculture, Chinese Academy of Sciences, Beijing, 100101, China

^3^ School of Agriculture, Ningxia University, Yinchuan, 750000, China

^4^Qi Biodesign, Beijing, 100101, China

^5^Key Laboratory of Potato Industry Integration and Development Enterprises in Inner Mongolia Autonomous Region, Hulunbuir, 021000, China

*** Correspondence:**

Pan Zhao

[zhaop@im.ac.cn](mailto:zhaop@im.ac.cn)

Supplementary Material

## Supplementary Figures


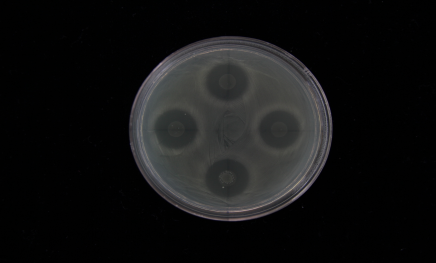

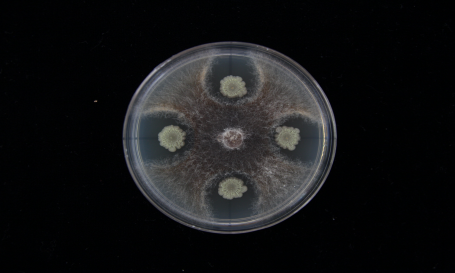

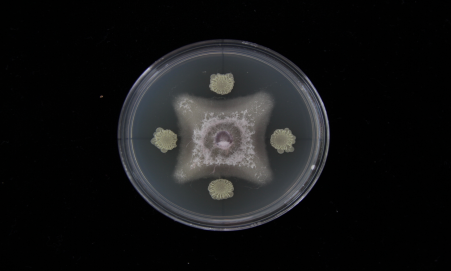

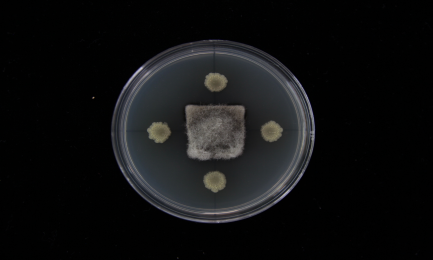

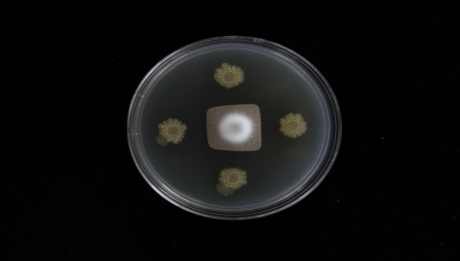

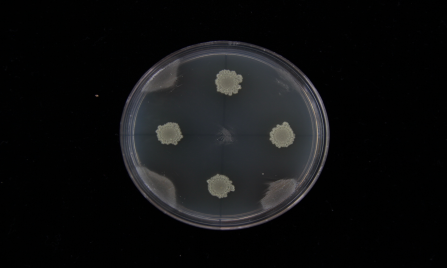


AA

A**A**

BA

CA

DA

EA

FA

**Supplementary Figure 1.** DX-9 had broad-spectrum antagonistic activity against different phytopathogens. **A** *Erwiniacarotovorasubsp.carotovora*. **B** *Rhizoctonia solani*. **C** *Fusarium oxysporum.* **D** *Alternaria solani.* **E** *Verticillium dahliae*. **F** *Streptomyces scabies*


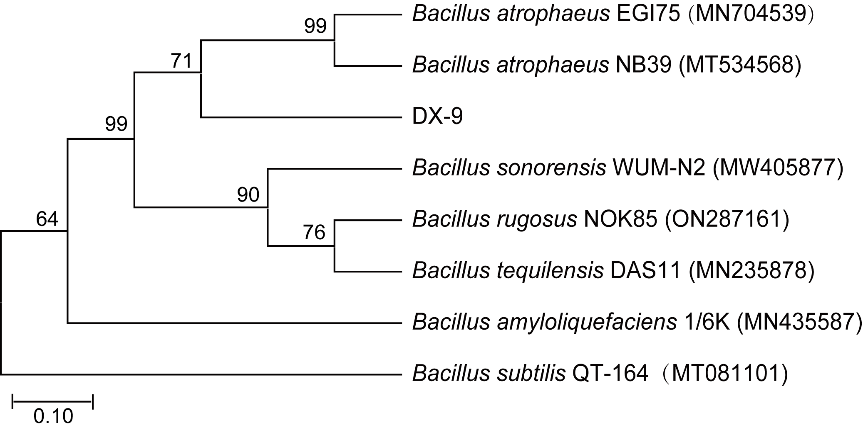


**Supplementary Figure 2.** Phylogenetic tree constructed based on 16S rDNA sequences of isolate DX-9.


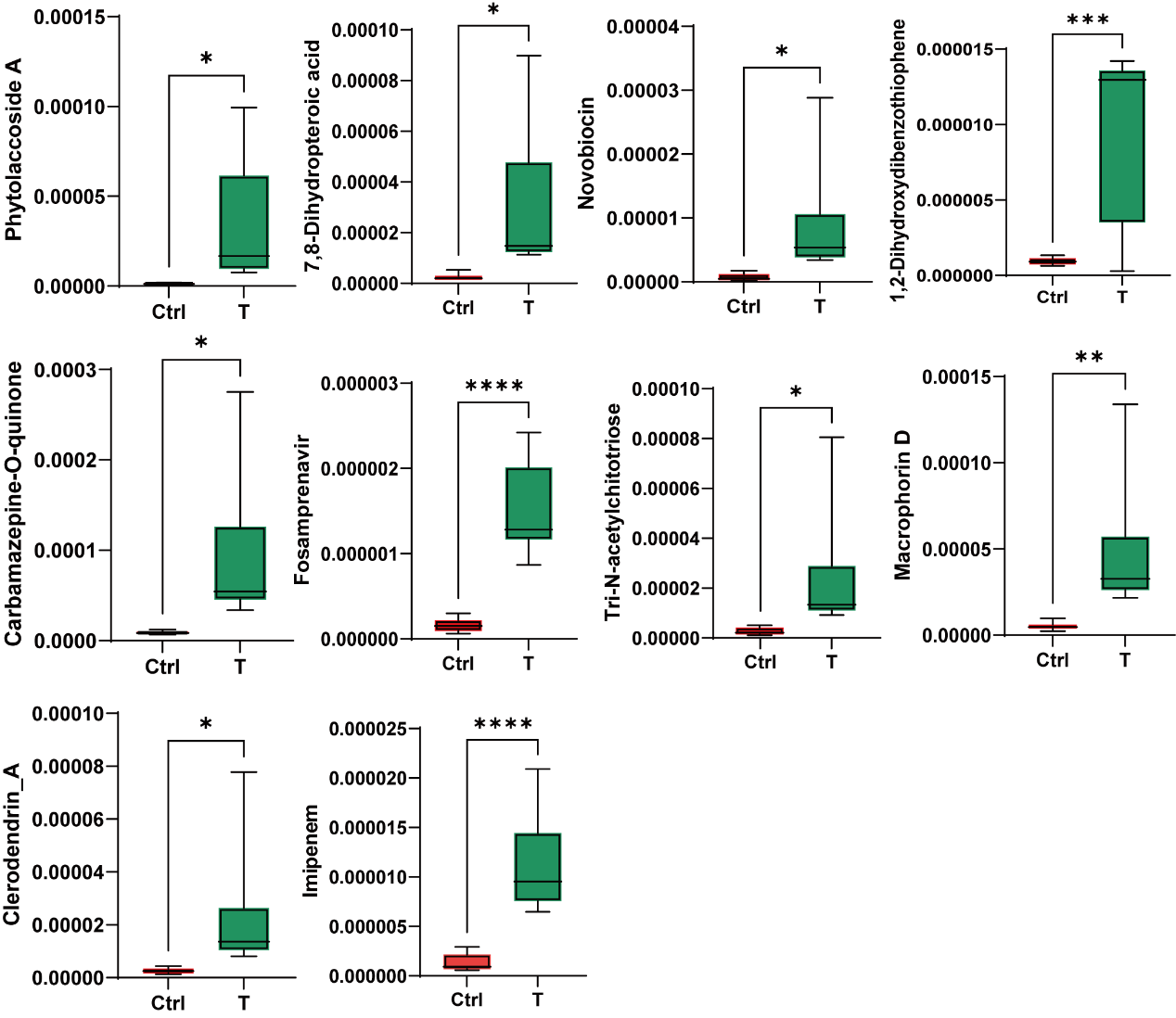


**Supplementary Figure 3.** The top 10 differential metabolites that relative content increased analysis for Ctrl vs T. * *P* < 0.05, ** *P* < 0.01, *** *P* < 0.001, based on Student’s *t*­test. Errors are the standard deviations (SDs) of means

**Supplementary Table 1.** Metabolism pathway annotation in different level by KEGG.

| Pathway ID | Name of pathway (level 3） | Name of pathway (level 2） | Name of pathway (level 1） | Count |
| --- | --- | --- | --- | --- |
| map04750 | Inflammatory mediator regulation of TRP channels | Sensory system | Organismal Systems | 1 |
| map04745 | Phototransduction - fly | Sensory system | Organismal Systems | 1 |
| map04742 | Taste transduction | Sensory system | Organismal Systems | 1 |
| map04728 | Dopaminergic synapse | Nervous system | Organismal Systems | 2 |
| map04725 | Cholinergic synapse | Nervous system | Organismal Systems | 1 |
| map04726 | Serotonergic synapse | Nervous system | Organismal Systems | 1 |
| map04723 | Retrograde endocannabinoid signaling | Nervous system | Organismal Systems | 3 |
| map04925 | Aldosterone synthesis and secretion | OrgEndocrine system | Organismal Systems | 1 |
| map04923 | Regulation of lipolysis in adipocytes | Endocrine system | Organismal Systems | 2 |
| map04916 | Melanogenesis | Endocrine system | Organismal Systems | 2 |
| map04614 | Renin-angiotensin system | Endocrine system | Organismal Systems | 1 |
| map04917 | Prolactin signaling pathway | Endocrine system | Organismal Systems | 2 |
| map04924 | Renin secretion | Endocrine system | Organismal Systems | 1 |
| map04977 | Vitamin digestion and absorption | Digestive system | Organismal Systems | 3 |
| map04978 | Mineral absorption | Digestive system | Organismal Systems | 6 |
| map04974 | Protein digestion and absorption | Digestive system | Organismal Systems | 8 |
| map04976 | Bile secretion | Digestive system | Organismal Systems | 7 |
| map04973 | Carbohydrate digestion and absorption | Digestive system | Organismal Systems | 2 |
| map04270 | Vascular smooth muscle contraction | Circulatory system | Organismal Systems | 1 |
| map04212 | Longevity regulating pathway - worm | Aging | Organismal Systems | 2 |
| map00351 | DDT degradation | Xenobiotics biodegradation and metabolism | Metabolism | 12 |
| map00364 | Fluorobenzoate degradation | Xenobiotics biodegradation and metabolism | Metabolism | 3 |
| map00791 | Atrazine degradation | Xenobiotics biodegradation and metabolism | Metabolism | 5 |
| map00623 | Toluene degradation | Xenobiotics biodegradation and metabolism | Metabolism | 8 |
| map00983 | Drug metabolism - other enzymes | Xenobiotics biodegradation and metabolism | Metabolism | 1 |
| map00626 | Naphthalene degradation | Xenobiotics biodegradation and metabolism | Metabolism | 5 |
| map00361 | Chlorocyclohexane and chlorobenzene degradation | Xenobiotics biodegradation and metabolism | Metabolism | 16 |
| map00621 | Dioxin degradation | Xenobiotics biodegradation and metabolism | Metabolism | 10 |
| map00624 | Polycyclic aromatic hydrocarbon degradation | Xenobiotics biodegradation and metabolism | Metabolism | 3 |
| map00365 | Furfural degradation | Xenobiotics biodegradation and metabolism | Metabolism | 1 |
| map00625 | Chloroalkane and chloroalkene degradation | Xenobiotics biodegradation and metabolism | Metabolism | 3 |
| map00633 | Nitrotoluene degradation | Xenobiotics biodegradation and metabolism | Metabolism | 3 |
| map00627 | Aminobenzoate degradation | Xenobiotics biodegradation and metabolism | Metabolism | 8 |
| map00643 | Styrene degradation | Xenobiotics biodegradation and metabolism | Metabolism | 1 |
| map00930 | Caprolactam degradation | Xenobiotics biodegradation and metabolism | Metabolism | 3 |
| map00362 | Benzoate degradation | Xenobiotics biodegradation and metabolism | Metabolism | 5 |
| map00363 | Bisphenol degradation | Xenobiotics biodegradation and metabolism | Metabolism | 1 |
| map00980 | Metabolism of xenobiotics by cytochrome P450 | Xenobiotics biodegradation and metabolism | Metabolism | 4 |
| map00982 | Drug metabolism - cytochrome P450 | Xenobiotics biodegradation and metabolism | Metabolism | 2 |
| map00642 | Ethylbenzene degradation | Xenobiotics biodegradation and metabolism | Metabolism | 1 |
| map00230 | Purine metabolism | Nucleotide metabolism | Metabolism | 5 |
| map01052 | Type I polyketide structures | Metabolism of terpenoids and polyketides | Metabolism | 6 |
| map01054 | Nonribosomal peptide structures | Metabolism of terpenoids and polyketides | Metabolism | 2 |
| map00908 | Zeatin biosynthesis | Metabolism of terpenoids and polyketides | Metabolism | 2 |
| map01057 | Biosynthesis of type II polyketide products | Metabolism of terpenoids and polyketides | Metabolism | 16 |
| map00981 | Insect hormone biosynthesis | Metabolism of terpenoids and polyketides | Metabolism | 2 |
| map00523 | Polyketide sugar unit biosynthesis | Metabolism of terpenoids and polyketides | Metabolism | 6 |
| map01059 | Biosynthesis of enediyne antibiotics | Metabolism of terpenoids and polyketides | Metabolism | 5 |
| map00900 | Terpenoid backbone biosynthesis | Metabolism of terpenoids and polyketides | Metabolism | 3 |
| map01055 | Biosynthesis of vancomycin group antibiotics | Metabolism of terpenoids and polyketides | Metabolism | 3 |
| map00906 | Carotenoid biosynthesis | Metabolism of terpenoids and polyketides | Metabolism | 17 |
| map00253 | Tetracycline biosynthesis | Metabolism of terpenoids and polyketides | Metabolism | 1 |
| map00904 | Diterpenoid biosynthesis | Metabolism of terpenoids and polyketides | Metabolism | 3 |
| map01053 | Biosynthesis of siderophore group nonribosomal peptides | Metabolism of terpenoids and polyketides | Metabolism | 2 |
| map00905 | Brassinosteroid biosynthesis | Metabolism of terpenoids and polyketides | Metabolism | 1 |
| map00522 | Biosynthesis of 12-, 14- and 16-membered macrolides | Metabolism of terpenoids and polyketides | Metabolism | 24 |
| map01051 | Biosynthesis of ansamycins | Metabolism of terpenoids and polyketides | Metabolism | 5 |
| map00450 | Selenocompound metabolism | Metabolism of other amino acids | Metabolism | 2 |
| map00410 | beta-Alanine metabolism | Metabolism of other amino acids | Metabolism | 2 |
| map00480 | Glutathione metabolism | Metabolism of other amino acids | Metabolism | 1 |
| map00460 | Cyanoamino acid metabolism | Metabolism of other amino acids | Metabolism | 7 |
| map00440 | Phosphonate and phosphinate metabolism | Metabolism of other amino acids | Metabolism | 5 |
| map00471 | D-Glutamine and D-glutamate metabolism | Metabolism of other amino acids | Metabolism | 1 |
| map00730 | Thiamine metabolism | Metabolism of cofactors and vitamins | Metabolism | 4 |
| map00860 | Porphyrin and chlorophyll metabolism | Metabolism of cofactors and vitamins | Metabolism | 7 |
| map00770 | Pantothenate and CoA biosynthesis | Metabolism of cofactors and vitamins | Metabolism | 3 |
| map00760 | Nicotinate and nicotinamide metabolism | Metabolism of cofactors and vitamins | Metabolism | 2 |
| map00740 | Riboflavin metabolism | Metabolism of cofactors and vitamins | Metabolism | 1 |
| map00790 | Folate biosynthesis | Metabolism of cofactors and vitamins | Metabolism | 2 |
| map00780 | Biotin metabolism | Metabolism of cofactors and vitamins | Metabolism | 1 |
| map00130 | Ubiquinone and other terpenoid-quinone biosynthesis | Metabolism of cofactors and vitamins | Metabolism | 8 |
| map00590 | Arachidonic acid metabolism | Lipid metabolism | Metabolism | 1 |
| map00561 | Glycerolipid metabolism | Lipid metabolism | Metabolism | 9 |
| map00072 | Synthesis and degradation of ketone bodies | Lipid metabolism | Metabolism | 1 |
| map00565 | Ether lipid metabolism | Lipid metabolism | Metabolism | 3 |
| map00140 | Steroid hormone biosynthesis | Lipid metabolism | Metabolism | 3 |
| map00592 | alpha-Linolenic acid metabolism | Lipid metabolism | Metabolism | 3 |
| map00121 | Secondary bile acid biosynthesis | Lipid metabolism | Metabolism | 2 |
| map00591 | Linoleic acid metabolism | Lipid metabolism | Metabolism | 4 |
| map00061 | Fatty acid biosynthesis | Lipid metabolism | Metabolism | 2 |
| map00073 | Cutin, suberine and wax biosynthesis | Lipid metabolism | Metabolism | 2 |
| map00564 | Glycerophospholipid metabolism | Lipid metabolism | Metabolism | 24 |
| map00120 | Primary bile acid biosynthesis | Lipid metabolism | Metabolism | 1 |
| map01040 | Biosynthesis of unsaturated fatty acids | Lipid metabolism | Metabolism | 5 |
| map00600 | Sphingolipid metabolism | Lipid metabolism | Metabolism | 1 |
| map00563 | Glycosylphosphatidylinositol (GPI)-anchor biosynthesis | Glycan biosynthesis and metabolism | Metabolism | 3 |
| map00550 | Peptidoglycan biosynthesis | Glycan biosynthesis and metabolism | Metabolism | 1 |
| map00540 | Lipopolysaccharide biosynthesis | Glycan biosynthesis and metabolism | Metabolism | 3 |
| map01230 | Biosynthesis of amino acids | Global and overview maps | Metabolism | 11 |
| map01220 | Degradation of aromatic compounds | Global and overview maps | Metabolism | 19 |
| map01120 | Microbial metabolism in diverse environments | Global and overview maps | Metabolism | 79 |
| map01210 | 2-Oxocarboxylic acid metabolism | Global and overview maps | Metabolism | 12 |
| map01110 | Biosynthesis of secondary metabolites | Global and overview maps | Metabolism | 55 |
| map01130 | Biosynthesis of antibiotics | Global and overview maps | Metabolism | 121 |
| map01100 | Metabolic pathways | Global and overview maps | Metabolism | 119 |
| map00920 | Sulfur metabolism | Energy metabolism | Metabolism | 2 |
| map00680 | Methane metabolism | Energy metabolism | Metabolism | 6 |
| map00190 | Oxidative phosphorylation | Energy metabolism | Metabolism | 1 |
| map00195 | Photosynthesis | Energy metabolism | Metabolism | 1 |
| map01060 | Biosynthesis of plant secondary metabolites | Chemical structure transformation maps | Metabolism | 13 |
| map01063 | Biosynthesis of alkaloids derived from shikimate pathway | Chemical structure transformation maps | Metabolism | 9 |
| map01066 | Biosynthesis of alkaloids derived from terpenoid and polyketide | Chemical structure transformation maps | Metabolism | 5 |
| map01070 | Biosynthesis of plant hormones | Chemical structure transformation maps | Metabolism | 5 |
| map01061 | Biosynthesis of phenylpropanoids | Chemical structure transformation maps | Metabolism | 5 |
| map01064 | Biosynthesis of alkaloids derived from ornithine, lysine and nicotinic acid | Chemical structure transformation maps | Metabolism | 3 |
| map01062 | Biosynthesis of terpenoids and steroids | Chemical structure transformation maps | Metabolism | 4 |
| map00052 | Galactose metabolism | Carbohydrate metabolism | Metabolism | 1 |
| map00040 | Pentose and glucuronate interconversions | Carbohydrate metabolism | Metabolism | 1 |
| map00650 | Butanoate metabolism | Carbohydrate metabolism | Metabolism | 1 |
| map00030 | Pentose phosphate pathway | Carbohydrate metabolism | Metabolism | 1 |
| map00520 | Amino sugar and nucleotide sugar metabolism | Carbohydrate metabolism | Metabolism | 5 |
| map00562 | Inositol phosphate metabolism | Carbohydrate metabolism | Metabolism | 1 |
| map00010 | Glycolysis / Gluconeogenesis | Carbohydrate metabolism | Metabolism | 2 |
| map00500 | Starch and sucrose metabolism | Carbohydrate metabolism | Metabolism | 2 |
| map00630 | Glyoxylate and dicarboxylate metabolism | Carbohydrate metabolism | Metabolism | 1 |
| map00940 | Phenylpropanoid biosynthesis | Biosynthesis of other secondary metabolites | Metabolism | 5 |
| map00960 | Tropane, piperidine and pyridine alkaloid biosynthesis | Biosynthesis of other secondary metabolites | Metabolism | 4 |
| map00942 | Anthocyanin biosynthesis | Biosynthesis of other secondary metabolites | Metabolism | 5 |
| map00404 | Staurosporine biosynthesis | Biosynthesis of other secondary metabolites | Metabolism | 7 |
| map00950 | Isoquinoline alkaloid biosynthesis | Biosynthesis of other secondary metabolites | Metabolism | 6 |
| map00311 | Penicillin and cephalosporin biosynthesis | Biosynthesis of other secondary metabolites | Metabolism | 5 |
| map00965 | Betalain biosynthesis | Biosynthesis of other secondary metabolites | Metabolism | 2 |
| map00525 | Acarbose and validamycin biosynthesis | Biosynthesis of other secondary metabolites | Metabolism | 4 |
| map00402 | Benzoxazinoid biosynthesis | Biosynthesis of other secondary metabolites | Metabolism | 1 |
| map00231 | Puromycin biosynthesis | Biosynthesis of other secondary metabolites | Metabolism | 5 |
| map00405 | Phenazine biosynthesis | Biosynthesis of other secondary metabolites | Metabolism | 3 |
| map00261 | Monobactam biosynthesis | Biosynthesis of other secondary metabolites | Metabolism | 6 |
| map00332 | Carbapenem biosynthesis | Biosynthesis of other secondary metabolites | Metabolism | 3 |
| map00901 | Indole alkaloid biosynthesis | Biosynthesis of other secondary metabolites | Metabolism | 4 |
| map00403 | Indole diterpene alkaloid biosynthesis | Biosynthesis of other secondary metabolites | Metabolism | 10 |
| map00524 | Neomycin, kanamycin and gentamicin biosynthesis | Biosynthesis of other secondary metabolites | Metabolism | 10 |
| map00401 | Novobiocin biosynthesis | Biosynthesis of other secondary metabolites | Metabolism | 6 |
| map00944 | Flavone and flavonol biosynthesis | Biosynthesis of other secondary metabolites | Metabolism | 1 |
| map00966 | Glucosinolate biosynthesis | Biosynthesis of other secondary metabolites | Metabolism | 12 |
| map00254 | Aflatoxin biosynthesis | Biosynthesis of other secondary metabolites | Metabolism | 1 |
| map00260 | Glycine, serine and threonine metabolism | Amino acid metabolism | Metabolism | 4 |
| map00380 | Tryptophan metabolism | Amino acid metabolism | Metabolism | 5 |
| map00270 | Cysteine and methionine metabolism | Amino acid metabolism | Metabolism | 3 |
| map00300 | Lysine biosynthesis | Amino acid metabolism | Metabolism | 1 |
| map00280 | Valine, leucine and isoleucine degradation | Amino acid metabolism | Metabolism | 2 |
| map00400 | Phenylalanine, tyrosine and tryptophan biosynthesis | Amino acid metabolism | Metabolism | 7 |
| map00350 | Tyrosine metabolism | Amino acid metabolism | Metabolism | 4 |
| map00330 | Arginine and proline metabolism | Amino acid metabolism | Metabolism | 2 |
| map00290 | Valine, leucine and isoleucine biosynthesis | Amino acid metabolism | Metabolism | 2 |
| map00220 | Arginine biosynthesis | Amino acid metabolism | Metabolism | 1 |
| map00360 | Phenylalanine metabolism | Amino acid metabolism | Metabolism | 6 |
| map00340 | Histidine metabolism | Amino acid metabolism | Metabolism | 3 |
| map05030 | Cocaine addiction | Substance dependence | Human Diseases | 2 |
| map05033 | Nicotine addiction | Substance dependence | Human Diseases | 1 |
| map05034 | Alcoholism | Substance dependence | Human Diseases | 3 |
| map05031 | Amphetamine addiction | Substance dependence | Human Diseases | 2 |
| map05032 | Morphine addiction | Substance dependence | Human Diseases | 1 |
| map05012 | Parkinson's disease | Neurodegenerative diseases | Human Diseases | 5 |
| map05020 | Prion diseases | Neurodegenerative diseases | Human Diseases | 1 |
| map05167 | Kaposi's sarcoma-associated herpesvirus infection | Infectious diseases: Viral | Human Diseases | 2 |
| map05145 | Toxoplasmosis | Infectious diseases: Parasitic | Human Diseases | 1 |
| map05143 | African trypanosomiasis | Infectious diseases: Parasitic | Human Diseases | 1 |
| map05150 | Staphylococcus aureus infection | Infectious diseases: Bacterial | Human Diseases | 1 |
| map05152 | Tuberculosis | Infectious diseases: Bacterial | Human Diseases | 1 |
| map05130 | Pathogenic Escherichia coli infection | Infectious diseases: Bacterial | Human Diseases | 2 |
| map01523 | Antifolate resistance | Drug resistance: Antineoplastic | Human Diseases | 2 |
| map05231 | Choline metabolism in cancer | Cancers: Overview | Human Diseases | 2 |
| map05230 | Central carbon metabolism in cancer | Cancers: Overview | Human Diseases | 7 |
| map05204 | Chemical carcinogenesis | Cancers: Overview | Human Diseases | 5 |
| map00970 | Aminoacyl-tRNA biosynthesis | Translation | Genetic Information Processing | 7 |
| map04080 | Neuroactive ligand-receptor interaction | Signaling molecules and interaction | Environmental Information Processing | 6 |
| map04022 | cGMP-PKG signaling pathway | Signal transduction | Environmental Information Processing | 1 |
| map04070 | Phosphatidylinositol signaling system | Signal transduction | Environmental Information Processing | 1 |
| map04024 | cAMP signaling pathway | Signal transduction | Environmental Information Processing | 3 |
| map04075 | Plant hormone signal transduction | Signal transduction | Environmental Information Processing | 2 |
| map04071 | Sphingolipid signaling pathway | Signal transduction | Environmental Information Processing | 2 |
| map02020 | Two-component system | Signal transduction | Environmental Information Processing | 2 |
| map02060 | Phosphotransferase system (PTS) | Membrane transport | Environmental Information Processing | 5 |
| map02010 | ABC transporters | Membrane transport | Environmental Information Processing | 14 |
| map07227 | Histamine H2/H3 receptor agonists/antagonists | Target-based classification: G protein-coupled receptors | Drug Development | 2 |
| map07218 | HIV protease inhibitors | Target-based classification: Enzymes | Drug Development | 1 |
| map07026 | Antifungal agents | Chronology: Antiinfectives | Drug Development | 1 |
| map07014 | Quinolones | Chronology: Antiinfectives | Drug Development | 1 |
| map07053 | Anti-HIV agents | Chronology: Antiinfectives | Drug Development | 1 |
| map04138 | Autophagy - yeast | Transport and catabolism | Cellular Processes | 3 |
| map04140 | Autophagy - animal | Transport and catabolism | Cellular Processes | 3 |
| map04136 | Autophagy - other | Transport and catabolism | Cellular Processes | 3 |
| map02024 | Quorum sensing | Cellular community - prokaryotes | Cellular Processes | 2 |
| map04217 | Necroptosis | Cell growth and death | Cellular Processes | 1 |
| map04111 | Cell cycle - yeast | Cell growth and death | Cellular Processes | 1 |

**Supplementary Table 2. The relative abundances of microorganisms that benefit to soil properties in different groups.**

| **Taxa** | **Ctrl** | **T** | **Fold Change** |
| --- | --- | --- | --- |
| *Enterobacter* | 891.84 | 2151.30 | 2.41 |
| *Salmonella* | 355.23 | 450.24 | 1.27 |
| *Flavobacterium* | 142.83 | 261.97 | 1.83 |
| *Rhizophagus* | 901.95 | 999.57 | 1.11 |
| *Aspergillus* | 309.30 | 360.84 | 1.17 |
| *Rhizobium* | 8071.41 | 8248.85 | 1.02 |
| *Bradyrhizobium* | 4408.70 | 5704.97 | 1.29 |
| *Agrobacterium* | 3423.75 | 3735.49 | 1.09 |
